# Percutaneous right ventricular assist device, rapid employment in right ventricular failure during septic shock

**DOI:** 10.1186/s13054-020-03413-4

**Published:** 2020-12-02

**Authors:** Ignazio Condello

**Affiliations:** Department of Cardiac Surgery, Anthea Hospital, GVM Care & Research, Via Camillo Rosalba 35/37, 70124 Bari, Italy

Right ventricular (RV) failure, defined by critical care echocardiography Right Ventricular dilatation) and a surrogate of venous congestion (Central Venous Pressure ≥ 8 mmHg), was frequently observed in septic shock patients and negatively associated with response to a fluid challenge despite significant pulse pressure variation (PPV) [1]. Right Heart Failure with invasive ventricular device is associated with significant morbidity and mortality. A new generation of percutaneous right ventricular assist devices (RVADs) may mitigate the need for invasive surgical RVAD implantation [2]. The Protek Duo (TPD) temporary RVAD, capable of providing up to 4.5 L/min [3], is a dual-lumen cannula inserted via the right internal jugular vein, with its proximal inflow lumen positioned in the right atrium and distal lumen positioned in the main pulmonary artery [4]. In this context, we read with interest the article: “Right ventricular failure in septic shock: characterization, incidence and impact on fluid responsiveness” by Vieillard-Baron A. and other authors. The article presents a multicenter care unit study about the incidence of RV failure on 282 patients with septic shock, its potential impact on the response to fluids, as well as tricuspid annular plane systolic excursion (TAPSE) values. Three groups of patients were compared according to RV size and CVP, a good surrogate of venous congestion. The study concluded that RV failure, defined by critical care echocardiography (RV dilatation) and a surrogate of venous congestion (CVP ≥ 8 mmHg), was frequently observed in septic shock patients and negatively associated with response to a fluid challenge despite significant PPV [1]. In this context, we propose for the ventilated septic shock patients with RV failure defined using critical care echocardiography (RV/LV end-diastolic area EDA ≥ 0.6) and a surrogate of venous congestion (CVP ≥ 8 mmHg), observed (42%) with a negative effect on the response to a fluid challenge, the rapid implantation of percutaneous right ventricular assist, with Protek Duo (TPD) device. The TPD weapon in association with or without membrane oxygenator (Fig. 1), with or without cytokine filters, could support: the right ventricular function hemodynamics, the ventricular recovery function, the pulmonary function and gas exchange, the barotrauma reduction, the fluid challenge response and containment of cytokine release syndrome. However, this approach may have a high impact in terms of healthcare costs, but we hypothesize it can significantly improve the outcome in the patients with right ventricular failure and septic shock patients, future studies should validate this technique and potential benefits.

**Fig. 1:**
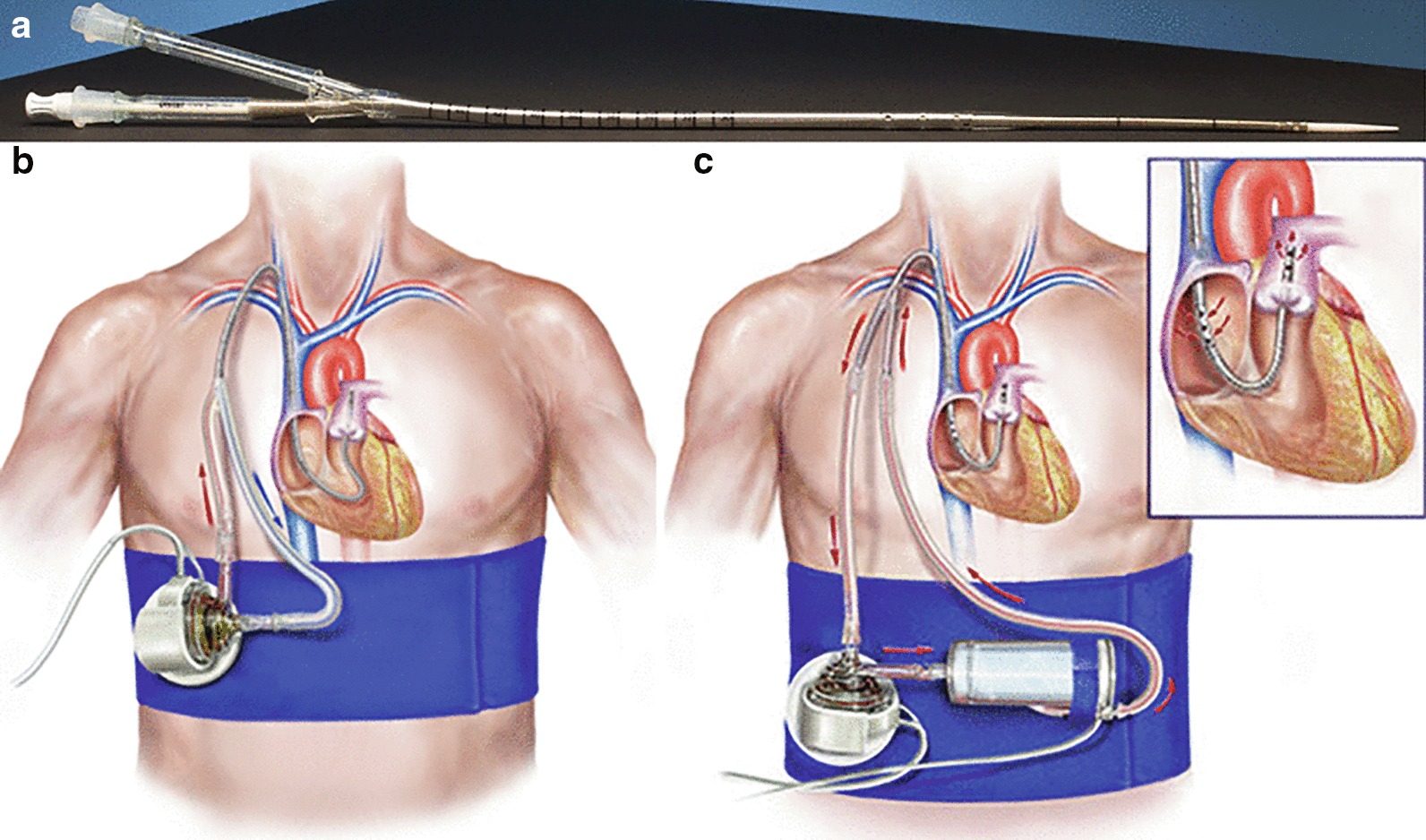
Percutaneous right ventricular assist device with **a** Protek Duo cannula, **b** without oxygenator, **c** with oxygenator

## Data Availability

Not applicable.

## References

[1] Vieillard-Baron A, Prigent A, Repessé X, Goudelin M, Prat G, Evrard B, Charron C, Vignon P, Geri G (2020). Right ventricular failure in septic shock: characterization, incidence and impact on fluid responsiveness. Crit Care.

[2] Salna M, Garan AR, Kirtane AJ, Karmpaliotis D, Green P, Takayama H, Sanchez J, Kurlansky P, Yuzefpolskaya M, Colombo PC, Naka Y, Takeda K (2020). Novel percutaneous dual-lumen cannula-based right ventricular assist device provides effective support for refractory right ventricular failure after left ventricular assist device implantation. Interact Cardiovasc Thorac Surg.

[3] Ravichandran AK, Baran DA, Stelling K, Cowger JA, Salerno CT (2018). Outcomes with the tandem Protek duo dual-lumen percutaneous right ventricular assist device. ASAIO J.

[4] Patel NJ, Verma DR, Gopalan R, Heuser RR, Pershad A (2019). Percutaneous biventricular mechanical circulatory support with impella CP and protek duo plus tandem heart. J Invasive Cardiol.

